# Challenges and potentialities of nursing work in street medical offices

**DOI:** 10.1590/1518-8345.2323.3045

**Published:** 2018-10-11

**Authors:** Aline Costa Cardoso, Débora de Souza Santos, Silvana Martins Mishima, Danielly Santos Cardoso dos Anjos, Jorgina Sales Jorge, Hiule Perreira de Santana

**Affiliations:** 1 Universidade Federal de Alagoas, Escola de Enfermagem e Farmácia, Maceió, AL, Brazil.; 2 Universidade Estadual de Campinas, Faculdade de Enfermagem, Campinas, SP, Brasil.; 3 Universidade de São Paulo, Escola de Enfermagem de Ribeirão Preto, PAHO/WHO Collaborating Centre for Nursing Research Development, Ribeirão Preto, SP, Brazil.

**Keywords:** Street People, Primary Health Care, Professional Practice, Nursing, Vulnerable Populations, Public Health

## Abstract

**Objective:**

to analyze elements of the nursing work process in the Street Medical Offices, highlighting the challenges and potentialities of care for homeless people.

**Method:**

this is an exploratory research of qualitative nature supported by the perspective of the health work process. The study was conducted through semi-structured interviews with nurses from the teams of the street medical offices at the city of Maceió and data were analyzed according to the content analysis technique, approaching issues related to the object, instruments and purposes of the nursing work process**.**

**Results:**

the identified themes were: Need for health care in the context of social and health vulnerability; Strategic planning and teamwork as tools for organizing the work process; Purposes and products of work: guaranteeing the right to access and care. . Before a work object designed by serious health needs resulting from the social vulnerability of this population, nurses use different instruments in their work process: strategic planning, acting in multiprofessional team and valorization of the light technologies of reception and bonding.

**Conclusion:**

apart from the difficulties, the study presents a successful experience that explores the potentiality of sharing relationships of humanized care.

## Introduction

With the increase in the number of homeless people in Brazil and at the same time initiatives such as the institution of the National Policy for the Homeless Population (PNHP), the public managers of the larger municipalities began to outline strategies to identify and approach the demands of this social group^1^.

The Unified Health System (SUS) represents a public system responsible for offering health actions and services in a universal, comprehensive and equitable way to the Brazilian population^2^. In 2011, the Ministry of Health of Brazil launched the National Policy on Primary Care in which Street Medical Offices (SMO) were established as one of its devices, aimed to attend the problems and needs of this social group and offer, in a more timely manner, health actions and services^1^.

It is incumbent on street medical office teams (SMOt) to pay attention to health in a differentiated and dynamic scenario that challenges health professionals who assist users in situations that do not fit the schedule. In order to overcome the difficulties, adversities and surprises found in this complex reality of the streets, the health work is organized in a multiprofessional and interdisciplinary perspective. In this context, it is expected that nursing, as part of the multiprofessional team, be able to maintain relationships that enable shared care, aiming at integral care of people in situations of extreme social vulnerability^3^.

In the context of the Brazilian Sanitary Reform and the change of the health care model in Brazil, some authors^4^ ^-^ ^6^ stood out in the discussion of the health work process and its role as mobilizer and constructor of new care arrangements.

The work in the health area is always a relational work^7^ because it depends on ‘live work’ in action, that is, a work performed at the moment it is (re)producing existences present in the act of care. These relations can be on the one hand brief and bureaucratic when care is centered in the prescriptive act, composing a model that has, in its essence, medical knowledge as hegemonic and producer of procedures, in which the dead work captures the living work.

For some authors^4^ ^-^ ^6^ ^,^ ^8^, health professionals understand the work process as a set of pieces of knowledge, instruments and means, having as subjects the professionals who organize themselves to produce services in order to provide individual and collective assistance to obtain products and results from their practice.

On the other hand, the nursing work aims to assist healthy or sick individuals, families and the community, performing care, management, and educational and investigative activities for the promotion, maintenance and recovery of individual and collective health^9^.

In this context, studying the nursing work process is important to understand the role of these workers and their work during the process, identifying their understanding about the object, the instruments used, the purpose and the final product obtained with the actions developed by the team and by nursing^10^.

Therefore, the care of the homeless population requires an increased focus on the health-disease-care process, as well as the use of various tools that value people and their needs, taking into account the territory and its singularities^1^ ^,^ ^3^ ^,^ ^11^.

Given the recent experience in Brazil of this public policy strategy and considering the fundamental role of nurses in the longitudinal follow up of this group that presents high social vulnerability, this study is justified by the fact that it brings to the fore a practice of nurses of major social relevance, focused on the qualification of care and the rescue of citizenship in the perspective of universal access to health services and health care. It should be noted that, in the process of preparing this article, we observed a scarcity of studies related to the work process in SMO and a lack of publications related specifically to nursing work in this context.

In this scenario, the study was conceived by the guiding question: do nurses have the potential to play an aggregating and articulating role in street care depending on how they organize their work process?

In order to elucidate this question, this article aimed to analyze the elements of the nursing work process in the SMO: the objects, the instruments and the purposes.

## Method

In order to achieve this objective, an exploratory research of a qualitative nature^12^ was carried out seeking the meaning of expression of ideas, feelings, values, and ways of thinking, relating and acting of nurses. This approach was considered the most adequate to capture the dynamics of the work process^5^ of these professionals in the context of the SMO, considering their constituent elements and the challenges and potentialities present in the daily care directed to people in situations of extreme social vulnerability.

The research scenario - a Street Medical Office in the municipality of Maceió - has 06 teams, classified as in the modality II. According to Ordinance nº 122, in this type of modality the team is formed by at least six professionals, among 03 with higher education and 03 with secondary education; the team may or may not have a medical professional and has the purpose of providing assistance for people living on the streets who are at personal and social risk, and whose attendance happens outside institutional walls. The SMO is part of the SUS’s Primary Health Care (PHC) service network and is articulated with other points of attention (outpatient and hospital units) in order to guarantee to users access to the system at all levels of complexity.

Five (05) nurses working in the SMO teams at the moment of data collection composed the participants of the study. The majority were female (04), with a mean age of 29 years and age range from 25 to 36 years. The criterion of inclusion of these subjects was having been working at the street medical office of Maceió for at least 6 months.

The technique used for data collection was semi-structured interviews conducted with aid of a script guided by questions rooted on the theoretical framework adopted. The questions turned to the constituent elements of the work process, namely: object, instruments, products and purpose.

Data were collected from June to July 2016 following all ethical procedures such as respect for the confidentiality of the participants and the consent to participate in the study by signature of the Informed Consent Term. The interviews were conducted individually in the reference units of each team at times agreed and chosen by the professionals. The interviews were recorded as a way to guarantee greater trustworthiness of the testimonies and had durations between 14m56s and 46m47s, being transcribed verbatim afterwards.

The treatment and interpretation of the data collected in the interviews were performed through the content analysis technique (12) in the thematic modality. The purpose of this analysis is to unravel the nuclei of meaning present in the communication, whose presence or frequency has some meaning for the object of study.

The steps of pre-analysis, material exploration, treatment of results and interpretation^12^ were followed. Thus, the following steps were performed: 1) data ordering, including the transcription, re-reading and organization of the reports; 2) then, categorization of the pieces of information after exhaustive and repeated reading of the texts for the definition of analytical categories; and 3) the third step consisted of the final analysis, which was based on the observed relationship between the empirical material (captured reality) and the theoretical reference on the health and nursing work process. In the set of empirical material, three themes emerged concerning the object of the work process, instrument and workforce, product and purpose of the work, defined as follows: “Need for health care in the context of social and health vulnerability”, “Strategic planning and teamwork as instruments for organizing the work process”; “Purposes and products of work: guaranteeing the right to access and care”. As already pointed out, all ethical criteria were respected and the study was approved on April 14, 2016, by the Ethics Committee of the Federal University of Alagoas under Opinion nº 1,500,689/CAAE nº52876016.6.0000.5013.

## Results

In the theme “Need for health care in the context of social and health vulnerability”, the strong context of vulnerability in which the users of the service are inserted was evidenced by the nurses of the SMO: *[...] First the basic needs that they do not have and whether we want or not it is health. Most of them have no guarantee of cleansing shower, no food guaranteed, all these things they have to go after them* (NUR 02).

When asked about the needs of users, the nurses highlighted the broad spectrum of needs encountered, especially when considering all the rights denied: *if we are to see them as integral subjects, they have several needs. Needs, for example, care needs with regard to nursing care that is our focus of attention, but they have need also related to relationships with other people, bonding with other people, need of being listened, need also of food because they are very needy, thus this is very [...]* (NUR 05).

Another health need identified was access to services of the Health Care Network (HCN): *there is a complex need, so to speak, because it requires a network. [...], the issue of the need to have housing, to have public security, to be part of the social assistance program and also of people in relation to health, the demands ... Scheduling of medical consultations, tests, other services* (NUR 04).

For workers, access to these devices would make them more likely to meet the needs of these users. However, this access is hampered by the lack of services and, especially, by a deficient reception: *[Regarding the difficulties] [...] problem is the gaze of some professionals, even the unit itself or other services that sometimes deny the service or do not understand. Because of a card of medical consultation… there have even been fights here already a few times* (NUR 01). *There is a lot of discrimination against these people in the service, I have already seen sad scenes of neglect and prejudice on the part of professionals who were attending this population in other hospital services* (NUR 04).

Health needs, as an object of nursing work, are typical of a dynamic and differentiated environment, requiring unique instruments and forms of organization of the work process by the nurses who experience the provision of street medical care, as discussed below.

In the second theme, “Strategic planning and teamwork as instruments for organizing the work process”, the following logic of work organization was identified: *we work in the following process: we have the pre- and post-field in the teams. So, in the pre-field, the whole team meets, determines the field that will be visited, the demands that are most frequent in that place and if something was left since the previous visit, so that we can already give an answer to the subject* (NUR 04). *[in relation to the organization of the work process] First, I mean, in a general way, there is already a process of team work. We do pre-field every day to leave to field, so we discuss the demands and we try to direct what we are going to solve or that audience that we are going to assist that day. And then planning is often not only the responsibility of nursing, it is with the participation of the team and, from that, we direct what we are going to do* (NUR 05).

We observed that the interviewed nurses print the characteristics of the Strategic Health Planning (SHP) in the organization of their work, characterizing it as a cyclical and systematized process for the identification, planning, execution and evaluation of health actions. The theoretical production of Mário Testa was used as reference for the SHP in this study, understanding it as a historical and social construction that takes place in the meeting and in the daily relations of power of health practices.

**Figure 1:**
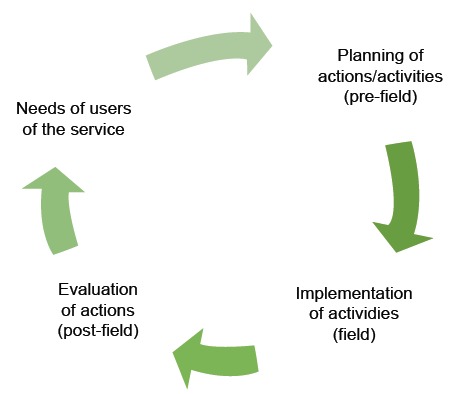
Diagram of the strategic planning of nurses in the Street Medical Office

To describe the SHP process, nurses use definition of the SMO, schematically presented below in Figure 1.

The nurses presented other instruments of their work process, such as supplies and equipment typical to nursing care (tensiometer, dressing materials, medications etc.). However, the use of light technologies received prominence, as a special aspect of good performance in SMO activities: *you bring this issue of instruments, but something that everyone ends up having to use is an appropriate language to speak to the person. So, you have to mix with people so that you can gain some confidence, because it is kind of... we’ll come with the car and just say good night and we want to start providing care as soon as possible; this would never happen, it never would work. We come closer trying to speak the same language in a very colloquial way sometimes, even using slang words, in the case, to get closer* (NUR 04).

And even using and valuing light technologies, nurses find it difficult to recognize them as work instruments, as evidenced in the following report: *then the first moment, to have a minimum functioning of the team, we must have this dialogue, this conversation. Then we check the pressure, do a blood glucose test, do prenatal monitoring, using sonar as well, of pregnant women on the streets. We use some tools that bring them closer and they see more results of the team action when there is something more concrete, when something is actually done on the street* (NUR 04).

It is noted, in the professionals’ speech, how this process becomes exhausting because they identify the population needs, carry out their planning, but find themselves in a situation of impotence before the management, when there is no guarantee that the resources and materials needed to carry out their planning will be available: *sometimes there is a shortage of resources as well, there is even a lack of transportation, some of the materials are also challenges for our work, because when we have a lack of material or lack of some resource or lack of transportation , sometimes we had an agenda to fulfill, a planning that we did and we often fail to meet that plan because of lack of transportation, for example, on that day* (NUR 05).

Before the lack of basic instruments of work, professionals end up investing in creativity and improvisation to overcome these situations, seeking to guarantee one of the primordial elements of their work: the bond with the users.

In the context of a work that is characterized by situational planning, valorization of relational technologies and creative efforts to overcome the structural deficiencies of street care, nurses point out a fundamental and integrating work element in this process, namely, the multiprofessional and interdisciplinary team: *[...] everyone gets involved in a way that is not one’s role, but we always end up sharing the work aiming the benefit of the user. It is interesting that we always share the demands* (NUR 03)*. Within the team, you have the specific academic training, but we actually work with interdisciplinarity, because we end up mixed a bit because of the needs in the medical office [...]* (NUR 04).

The third theme concerns the “ Purposes and products of work: guaranteeing the right to access and care”. At first glance, nurses point out the users’ autonomy as the primary purpose of their work: *[In relation to the purpose of the work] So, we try to incite them to action, to see if they have that autonomy* (NUR 01).

For nurses, seeking the autonomy of the users is related to other purposes of their work: improving the quality of life and guaranteeing the right of access to health services: *[Identified as the purpose of their work] So, we try to guarantee the performing, that they really have this right of access and also that they have, as I have already said, a better quality of life, [...] sometimes, because they do not believe in life anymore, because there are people who we have already assisted in such a critical situation and after that we started to do the follow up and we noticed the changes* (NUR 05).

Another purpose of the work in SMO highlighted by the nurses was the improvement of the quality of life. Of course, this is one of the most difficult tasks, considering all the complex context of needs and deprivations to which this population is subjected: *[...] in general, the purpose of our work, what do we expect? That these people have a better quality of life, regardless if they will be on the street, if they go back to the family, go to some institution. So, the goal of our work is to encourage them to have a better life, to get more pleasure in some of the activities they do on a daily basis, to recognize and to realize that sometimes that people want to change or want an opportunity too and they often do not know how to do or they do not have the support of anyone* (NUR 05).

In order to seek quality of life within the possible and actual context, nurses of the SMO highlighted harm reduction (HR) also as the purpose of their work: *[in relation to harm reduction] (...) but you know how to work harm reduction with them so that they gradually understand what is best for their health, right? “”Oh, if you smoke 40 cigarettes, smoke 38 at least “ (laughs) and you keep decreasing* (NUR 03)*.*

## Discussion

In the light of the analytical themes emerging from the nurses’ speeches in this study, and when we focus specifically on the nursing work process, we have as object of work the “care” or “needs” of individuals, families, communities and collectivities. It is understood that the object of the health and nursing work is not limited to the physical/biological body of these individuals. This conception vary according to the conception of health and disease with which one is dealing^13^.

Thus, identifying the health needs of the users requires overcoming the disease-centered paradigm as the only means of dealing with the complaints presented by users, representing their health problems. Health needs are not restricted simply to human biological aspects, but rather encompass the needs and vulnerabilities of the individuals^14^.

Another need that was highlighted in the interviewees’ speeches is the access to health services. This finding is ratified by the literature, because health needs also imply access to services and technologies that prolong or improve life, including the necessary referrals, as well as the scheduling of consultations in other points of the HCN^14^ ^-^ ^15^.

Access to the HCN has also been discussed by authors^1^ ^,^ ^3^ ^,^ ^11^ who point out the process of isolation that street office teams suffer in relation to the Network and how this impacts on the difficulty of access of the users. The professionals would be “imprisoned” in an inflexible routine to deal with different, unpredictable situations, being this model incompatible with the work process in the SMO.

Another aspect observed was the transformative potential of the work of nurses in SMO and the care offered, since these are guided by light technologies such as bonding, embracement, sensitive listening and empathy. In this perspective, care is based on the sharing of experiences and the different ways of intervening, considering the subjectivity and the valorization of the others in order to favor the strengthening of their self-esteem and autonomy for self-care.

Confronted with the statement of NUR 04, we observed that colloquial language is used in the communication with the users as a strategy aiming at the approximation and establishment of a relationship of trust and respect, essential for the construction of the technology called “bond”. Bond is considered a light technology^4^ ^,^ ^16^, or relational technology, that precedes any other action of care, because it is through it that the professionals can establish a relationship of trust with others. Consequently, there is a greater likelihood of achieving therapeutic success by means of greater adherence to a particular treatment, changing habits, or even establishing new ways of developing self-care.

Historically, the formation of the hegemonic medical-assistance model^4^ ^,^ ^17^ was centered on hard and light-hard technologies as it was based on corporate interests, especially of economic groups^18^. With regard to the micropolitical organization of the health work, this model has produced a work organization with a flow directed to medical consultations, in which the medical knowledge structures the work of other professionals, and the production of care ends up dependent on these more structured instruments.

In the face of this paradigm, of overvaluation of hard and light-hard technologies, usually even when the professionals identify within the work process the use of light technologies as guiding tools, they may believe that their work is only recognized through use of hard technologies.

The users’ needs to make this care objectified into something “concrete”, through hard technologies, seems to translate care in a more visible way, and it is up to the professionals to balance the use of the various instruments of their work.

Still with regard to the instruments, we observed in the speeches the lack of some essential supplies and materials and how this reflects in the quality of the service provided to the users in the view of the professionals. Management has left a gap with respect to the discontinuation of the supply of material for care provision. The lack of resources has a negative effect even on the bonding and trust built with users.

Similar results were found in a study^1^ ^)^ performed with SMO teams in the city of Rio de Janeiro in 2013, in which the professionals indicated to work based on a perspective of shared care.

It is important to remember that health, driven and legitimized by the hegemonic care model, has undergone a strong fragmentation process due to the phenomenon of professional super-specialization and increasing technical and social division of health work^4^ ^,^ ^17^ ^-^ ^18^.

In the context of model change, the ever-increasing need for new and complex tasks to reach a comprehensive health care is added to this panorama, requiring from professionals the capacity of interdisciplinary teamwork^15^ ^,^ ^17^.

The promotion of integrated teamwork aims to improve service responses to users’ needs and the quality of health care^19^. This requires, therefore, collaboration between professionals from different areas with a focus on the needs, as happens in the reality of the SMO investigated.

It can then be inferred that the exercise of teamwork is one of the potentialities of subjects in the work organization in the scenario investigated, often being the main instrument available for overcoming the barriers and daily difficulties of workers. Among these, the difficulty of access makes up the context of exclusion of the homeless population.

According to the interviewed nurses, the difficulty that the homeless people face in accessing the HCN is mainly related to the lack of preparation on the part of professionals who make up the network services, who often manifest prejudice and resistance to provide care for these people. This process of exclusion discourages the SMO nurses who often works hard to establish a relationship of bond and trust with the users, and this bond is sometimes broken by unprepared professionals from other points of the HCN (Health Care Network).

Quality of life as a health promotion tool ^20^ encompasses, in addition to health status, satisfactory relationships with the environment, economic resources and time for work and leisure. Considering that the homeless population is marked by difficult living conditions, poor food and housing, besides lack of access to basic rights such as education, health and safety, it is understood that quality of life is sought in a more real and contextualized perspective, to provide more dignity to life^3^.

In this sense, efforts have been made to conquer spaces, as well as to establish a relationship between quality of life and social justice, so that the focus may not only be on reducing the risks of diseases, but also increasing life opportunities, for which progressive multi- and inter-sectorial articulation is required^15^. This has been pointed out as one of the greatest barriers found, not only by SMO team workers of the northeastern capital, but also in other states, as described in studies already cited^1^ ^,^ ^3^ ^,^ ^11^.

The right to health, therefore, is signaled at all times as the primary purpose of the work process in SMO, understood as a set of minimum goods and services necessary for the survival and possibility of improving the quality of life, as established in article 25 of the Universal Declaration of Human Rights^21^. The discussion of the interface between universal human rights and health has mobilized national and international authors in a common understanding of favoring the empowerment of both users and health workers in the knowledge about human rights and citizenship, developing strategies that first ensure the interest of the human beings in dialogical and reciprocal actions of solidarity^22^ ^-^ ^23^. In Brazil, this movement has been expanded with the National Health Humanization Policy, focusing on changes in health practices that value the life and the participation of people in care processes^24^.

In this perspective, the damage reduction (DR) was pointed out by the nurses as well as a purpose of their work process. Damage reduction was initially understood as an initiative aimed at controlling the consequences of the use of psychoactive substances, without necessarily imposing it on abstinence^1^. It is noteworthy that the nurses participating in this study, although facing difficulties of different orders in their work process, defined the purposes of their work in a way that is aligned with the current care policies and strategies for homeless people who commonly make use of psychoactive substances.

The analysis of the nursing work process in the SMO allowed, in the distinction and reflection about their objects, instruments and purposes, to glimpse the potentialities of the practice of nurses in this context, which at the same time qualifies the care for homeless people and also potentialities the very essence of the nursing practice^9^: the care. Among these potentialities, the following should be highlighted: the broadening of the look at health problems and needs, the exercise of teamwork, the emphasis on light care technologies, and the commitment to guarantee the right to health to all citizens.

## Conclusion

The investigation of the constituent elements of the nursing work process at the Street Medical Office provided several reflections about the ways of caring for homeless populations and how professionals deal with the complexity of working outside conventional settings.

The study presented some limitations typical of exploratory qualitative researches: it was carried out in a single municipality, comprising a small number^5^ of interviewed subjects, which represented at the time of field research all the nurses working in the CnaR of the municipality in question. Despite these limitations, the study met its objective and provided answers, by examining the constituents of the work process, about the potentiality of the nursing practice to improve street care.

Regarding the object of work, a broad spectrum of care needs was highlighted, including issues that emanate from the context of social vulnerability and that translate into needs of food, hygiene, housing and education, to issues of diagnostic and therapeutic intervention, highlighting the need for access to the Health Care Network.

To face with this reality, the nurses use instruments of different technological densities, with emphasis on strategic planning, acting in an interdisciplinary team, and valuing the light technologies such as embracement and bond in the relationship of care with the users. The configuration of this work process begins and ends with the central purpose of guaranteeing the right of this population to have access to health, to improve the quality of life and to live with more autonomy.

Despite the difficulties faced by nurses in their work in SMO, the study presents a successful experience that explores the potentiality of caring relationships of humanized care. These tools are essential to achieve one of the main purposes of their work, to help homeless people exercise their autonomy and build the protagonism of their own care despite so many adversities.

As challenges, the research indicates the need for permanent investments, in both material and human resources, by health management, to qualify the work routine of the SMO, and to broaden the understanding of the various actors of the Health Care Network about the purpose of the work done with homeless people.

Because this is not a work that is limited to the hands and voices of the professionals of this device, but it is rather extended to actions that require co-responsibility and sensitivity of different points and sectors of the public service network, with a view to the extension of the rights of a population that already suffers so much and is forgotten in the urban centers in Brazil.

## References

[1] Engstrom EM, Teixeira MB (2016). Manguinhos, Rio de Janeiro, Brazil, “Street Clinic” team: care
and health promotion practice in a vulnerable territory. Ciênc Saúde Coletiva.

[2] Andrade FR, Narvai PC (2013). Population surveys as management tools and health care
models. Rev Saúde Pública.

[3] Londero MP, Ceccim RB, Bilibio LFS (2014). Consultation office of/in the street: challenge for a healthcare
in verse. Interface. (Botucatu).

[4] Carvalho BG, Peduzzi M, Mandú ENT, Ayres JRCM (2012). Work and Inter-subjectivity: a theoretical reflection on its
dialectics in the field of health and nursing. Rev. Latino-Am. Enfermagem.

[5] Silva KL, Sena RR, Seixas CT, Feuerwerker LCM, Merhy EE (2010). Home care as change of the technical-assistance
model. Rev Saúde Pública.

[6] Slomp JH, Feuerwerker LCM, Merhy EE (2015). Life stories, homeopathy and permanent education: construction of
shared healthcare. Ciênc Saúde Coletiva.

[7] Chagas MS, Abrahão AL (2016). Care production in health team focused on living work: the
existence of life on death territory. Interface. (Botucatu).

[8] Cavalcante MDMA, Larocca LM, Chaves MMN, Cubas MR, Piosiadlo LCM, Mazza VA (2016). Nursing terminology as a work process instrument of nurses in
collective health. Rev Esc Enferm USP.

[9] Matumoto S, Fortuna CM, Kawata LS, Mishima SM, Pereira MJB (2011). Nurses’ clinical practice in primary care: a process under
construction. Rev. Latino-Am. Enfermagem.

[10] Souza IAS, Pereira MO, Oliveira MAF, Pinho PH, Gonçalves RMDA (2015). Work process and its impact on mental health nursing
professionals. Acta Paul Enferm.

[11] Alecrim TFA, Mitano F, Reis A, Roos CM, Palha PF, Protti-Zanatta ST (2016). Experience of health professionals in care of the homeless
population with tuberculosis. Rev Esc Enferm USP.

[12] Minayo MCS (2012). Qualitative analysis: theory, steps and
reliability. Ciênc Saúde Coletiva.

[13] Agreli HF, Peduzzi M, Silva MC (2016). Patient centred care in interprofessional collaborative
practice. Interface. (Botucatu).

[14] Hino P, Takahashi RF, Bertolozzi MR, Villa TCS, Egry EY (2012). Family health team knowledge concerning the health needs of
people with tuberculosis. Rev. Latino-Am. Enfermagem.

[15] Moraes PA, Bertolozzi MR, Hino P (2011). Perceptions of primary health care needs according to users of a
health center. Rev Esc Enferm USP.

[16] Santos FPA, Nery AA, Matumoto S (2013). Care provided to patients with hypertension and health
technologies for treatment. Rev Esc Enferm USP.

[17] Viegas SMF, Penna CMM (2015). Integrality: life principle and right to health. Invest Educ Enferm.

[18] Iriart C, Merhy EE (2017). Inter-capitalistic disputes, biomedicalization and hegemonic
medical model. Interface. (Botucatu).

[19] Peduzzi M (2013). Interprofessional education: training for healthcare
professionals for teamwork focusing on users. Rev Esc Enferm USP.

[20] Tavares MFL, Rocha RM, Bittar CML, Petersen CB, Andrade M (2016). Health promotion in professional education: challenges in Health
and the need to achieve in other sectors. Ciênc Saúde Coletiva.

[21] Assembly, Nations General (1948). “Universal Declaration of Human Rights.”.

[22] Stefanini A, Ziv H (2004). Occupied Palestinian Territory: Linking Health to Human
Rights. Health Hum Rights.

[23] Mann J (2011). Health and Human Rights. Am J Public Health.

[24] Dalla NCR, Junges JR (2013). Humanization policy in primary health care: a systematic
review. Rev. Saúde Pública.

